# Cerebral blood volume and oxygen supply uniformly increase following various intrathoracic pressure strains

**DOI:** 10.1038/s41598-017-08698-0

**Published:** 2017-08-21

**Authors:** Zhongxing Zhang, Nina Bolz, Marco Laures, Margit Oremek, Christoph Schmidt, Ming Qi, Ramin Khatami

**Affiliations:** 1 Center for Sleep Medicine and Sleep Research, Clinic Barmelweid AG, Barmelweid, Switzerland; 2 Department of Neurology, Inselspital, Bern University Hospital, University of Bern, Bern, Switzerland; 3 Cardiac Rehabilitation Clinic, Clinic Barmelweid AG, Barmelweid, Switzerland

## Abstract

Intrathoracic pressure (ITP) swings challenge many physiological systems. The responses of cerebral hemodynamics to different ITP swings are still less well-known due to the complexity of cerebral circulation and methodological limitation. Using frequency-domain near-infrared spectroscopy and echocardiography, we measured changes in cerebral, muscular and cardiac hemodynamics in five graded respiratory maneuvers (RM), breath holding, moderate and strong Valsalva maneuvers (mVM/sVM) with 20 and 40 cmH_2_O increments in ITP, moderate and strong Mueller maneuvers (mMM/sMM) with 20 and 40 cmH_2_O decrements in ITP controlled by esophageal manometry. We found cerebral blood volume (CBV) maintains relative constant during the strains while it increases during the recoveries together with increased oxygen supply. By contrast changes in muscular blood volume (MBV) are mainly controlled by systemic changes. The graded changes of ITP during the maneuvers predict the changes of MBV but not CBV. Changes in left ventricular stroke volume and heart rate correlate to MBV but not to CBV. These results suggest the increased CBV after the ITP strains is brain specific, suggesting cerebral vasodilatation. Within the strains, cerebral oxygen saturation only decreases in sVM, indicating strong increment rather than decrement in ITP may be more challenging for the brain.

## Introduction

Moderate and strong intrathoracic pressure (ITP) swings frequently occur voluntarily and involuntarily in daily life (e.g., defecation, diving, heavy weightlifting and coughing), and during positive airway pressure therapies in patients (e.g., patients with sleep apnea or respiratory failure in critical care). ITP swings challenge many physiological systems^1–5^. The hemodynamic consequences under different ITPs are of major interest for scientists and clinicians. Currently, our knowledge is mainly from systemic and peripheral hemodynamics (e.g., heart rate (HR), left ventricular stroke volume (LVSV), blood pressure, peripheral vascular resistance^1, 2, 6–8^). How ITP changes influence cerebral hemodynamics (CH) which is regulated by cerebral autoregulation (CA) is still less known.

CA is a complex physiological process counteracting the cerebral perfusion pressure (CPP) changes to maintain cerebral blood flow (CBF) and oxygenation. CPP is the difference between mean arterial pressure (MAP) and intracranial pressure (ICP), and usually dominated by MAP because ICP is much smaller compared to MAP. Thus normally the function of CA can be checked by measuring changes in CBF under the manipulation of MAP^9^. However, this regular approach of examining CA may not work when ITP changes because strong ITP swings are directly transmitted to ICP and considerably influence CPP^3, 10^. Changes in cardiac output and central blood volume (BV) also influence the function of CA^11^. These aforementioned factors make it more demanding to investigate cerebral hemodynamics during ITP swings. Methodological limitation is another issue hindering the study of dynamic cerebral autoregulation and hemodynamics under different ITPs. Functional magnetic resonance imaging (fMRI) cannot be integrated with echocardiography, so its application is limited in this topic as it cannot explore the interactions between the CH and systemic hemodynamics. Transcranial Doppler (TCD) can only assess blood flow velocity and its reliability is based on the assumption of unchangeable vessel diameter^12^, which is not longer valid under hypercapnia/hypoxia. Besides, keeping a precise location of the TCD probe during measurement is usually difficult^9, 11, 13^.

Near-infrared spectroscopy (NIRS) is an optical technique that can quantify changes in oxygenated hemoglobin (HbO_2_), deoxygenated hemoglobin (HHb), tissue oxygen saturation (StO_2_), oxygen index (OI, HbO_2_-HHb) and total hemoglobin (HbO_2_ + HHb) in both local cerebral and muscular tissues^14–16^. Total hemoglobin measured by NIRS is suggested to be a surrogate index reflecting the change in local BV, which can directly characterize perfusion and vasomotor activities (vasodilatation/vasoconstriction) in local tissue^17–20^. This technique holds the promise to be a proper technique assessing changes in multi-component of cerebral hemodynamics under various ITPs in both basic and clinical research^9, 20–24^. However, the reliability of NIRS measurement has been debated for decades. Previous studies repeatedly reported that the cerebral hemodynamics measured with NIRS are contaminated by extracranial tissues^25, 26^, even the ones measured with NIRS devices that received USA Food and Drug Administration’s approval^27^. The results of NIRS studies investigating CA and CH are questioned^28^. These negative results made clinicians and scientists hesitate to use this technique. However, it is important to notice that the negative results published were measured with continuous wave (CW) NIRS, a relative simple technique applying modified Beer-Lambert law (MBLL) to calculate hemodynamics^15^. Frequency-domain multi-distance (FDMD) NIRS is an advanced technique that is less affected by the superficial influences compared to CW NIRS^15, 29^. Its reliability of measuring the cerebral hemodynamic changes has been validated in invasive animal study^30^, and in non-invasive human cognitive studies with simultaneous fMRI measurements^31, 32^.

Here we study the changes in cerebral, muscular and cardiac hemodynamics induced by five different respiratory maneuvers (RMs) (i.e., breath holding (BH), moderate and strong Mueller maneuvers (mMM and sMM), and moderate and strong Valsalva maneuvers (mVM and sVM)) using FDMD NIRS and echocardiography in healthy subjects. These RMs are well-established physiological models used in basic and clinical research, and routine physical and medical examinations (e.g., MM and BH are frequently used to mimic obstructive and central sleep apnea events^2, 6, 7, 33^; VM is a routine medical test for cardiac functions^5^). To our knowledge, this is the first study comprehensively characterizing the changes in CH challenged by different ITPs (i.e., ITP changes in different directions and degrees), with peripheral (i.e., muscular) and systemic (i.e., cardiac) hemodynamics as controls. We hypothesize that: 1). cerebral BV (CBV) should show different changes compared to muscular BV (MBV), given that measured CBV really reflects changes in the brain. 2). CBV may keep stable in each RM reflecting intact CA in healthy subjects, and changes in MBV, HR and LVSV may be variable.

## Results

### Changes in CBV and cerebral NIRS signals

**Figure 1:**
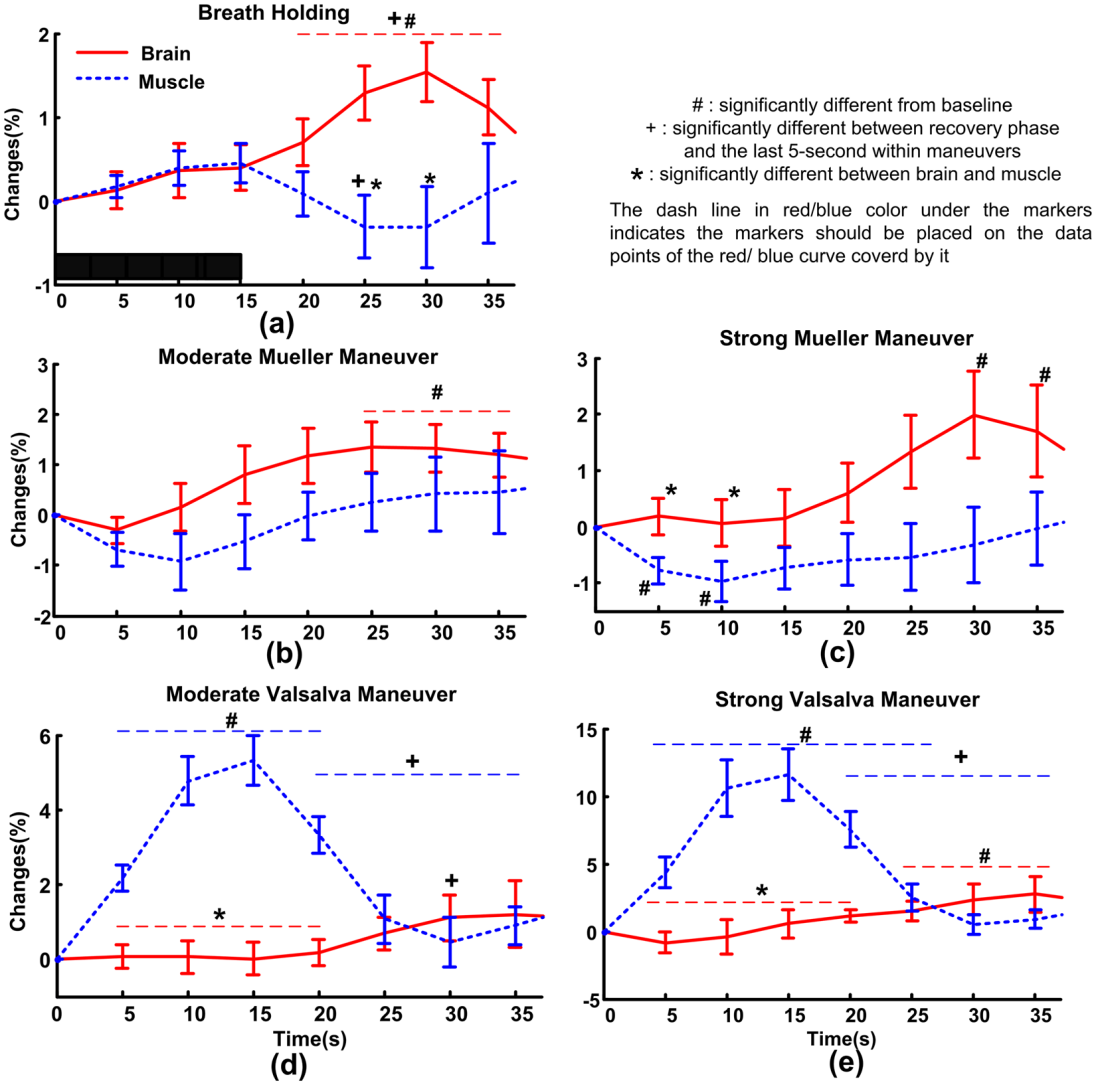
The mean relative changes of blood volume in brain and muscle in each maneuver. The black bar covering 0 to 15 s in (**a**) indicates the period within strain. The error bars are standard error and n = 12.

The linear mixed model (LMM, random slope model, considering that the NIRS signals are normalized to the baseline) fitted slopes of CBV changes versus time in BH, mMM, sMM, mVM and sVM are 0.21, 0.24, 0.3, 0.19 and 0.5, and they are statistically significant (p-values are < 0.0001, 0.003, 0.0025, 0.043 and 0.0011, respectively), suggesting that CBV is generally monotonically increasing in each RM protocol. CBV mainly increases in the recovery phase and it is not significantly different from its baseline within the strains (Paired t-tests, Fig. 1). After false discovery rate (FDR) control, the increment of CBV during recovery phase is still significant in BH and tends to reach statistical significance in mMM, sMM, mVM and sVM (p-value increases to 0.05, 0.1, 0.1 and 0.1, respectively). The results of LMMs and t-test suggest that the changing patterns of CBV should hold in spite of slight increase of p-values after FDR, i.e., CBV slowly but steadily increases after ITP strains.

**Figure 2:**
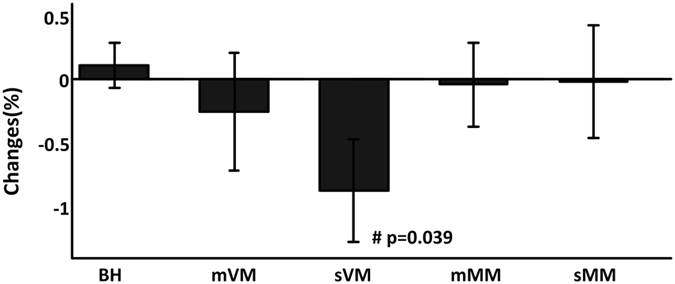
Relative changes of cerebral StO_2_ in the last 5 s maneuvers compared to its baseline level. ^#^Indicates significant differences. The error bars are standard error and n = 12.

The stable CBV within the strain may indicate the function of CA maintaining adequate blood and oxygen supply. To test this hypothesis we use Paired t-test to compare the changes of cerebral StO_2_ in the stable last 5 s of RM to its baseline. Significant decrement is only observed in sVM (Fig. 2).

**Figure 3:**
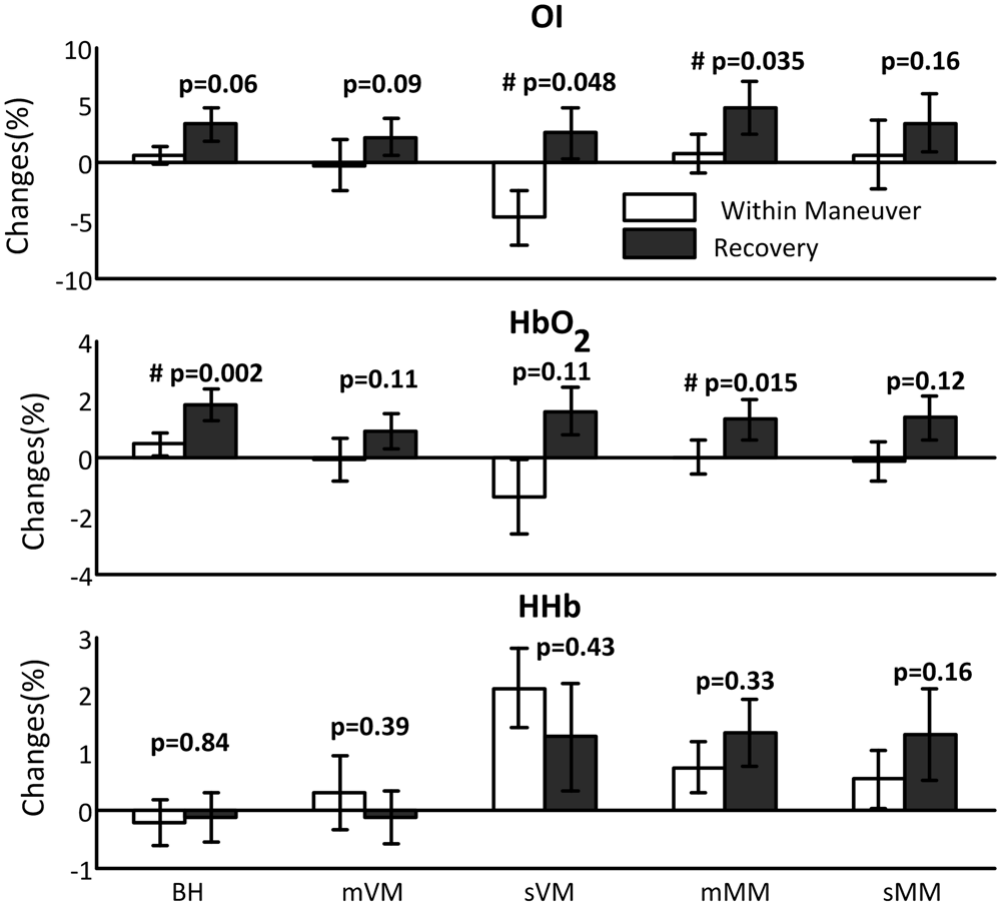
The changes in cerebral oxygen index (OI), HbO_2_ and HHb between the periods within maneuver and recovery. ^#^Indicates significant differences. The error bars are standard error and n = 12.

To explore the changes in other hemodynamics associated with increasing cerebral perfusion post strain we compare their mean changes within maneuver to the ones within 15 s recovery period (Paired t-test, Fig. 3). Cerebral OI and/or HbO_2_ significantly increase in recovery phase, except for sMM and mVM in which the increments tend to reach statistical significance. HHb shows no significant change.

### Changes in MBV

The main effect of changes in MBV shows only non-significant trends (the fitted LMM slopes of MBV versus time in BH, mMM, sMM, mVM and sVM are −0.05, 0.15, 0.04, −0.18 and −0.49; p-values are 0.429, 0.149, 0.639, 0.129 and 0.075), due to the non-monotonic changes of MBV in the whole RM protocol (Fig. 1). MBV significantly increases within the strain and decreases to baseline level in the recovery period in VM (Paired t-tests, Fig. 1(e,f)). Significant decrement is found within the stain of sMM (Fig. 1(c)) and in the recovery phase of BH (Fig. 1(a)). Within the stain of mMM, MBV decreases in the first 5 s of RM and tends to reach statistical significance (p = 0.068). After FDR control these changes are still significant, except for the decrement of BV after BH (p-value increases to 0.1, which implies a trend to significance).

### The relationship between changes in CBV, MBV and ITP

The changes of CBV and MBV are significantly different in all RMs (p-values of categorical variable ‘tissue’ fitted by LMM in all RMs are smaller than 0.0001). Paired t-tests find significant differences between CBV and MBV within the recovery phase of BH, mVM and sVM, and within the strain of sMM, mVM and sVM (Fig. 1). These differences are still significant after FDR control.

The mean changes in ITP are 1.16 ± 6.71, 22.58 ± 7.93, 43.65 ± 8.54, −28.83 ± 11.9, −52.49 ± 12.9 (mean ± standard deviation) cmH_2_O in BH, mVM, sVM, mMM and sMM, respectively. The relative large standard deviation suggests large individual differences. But the changes in ITP can be clearly graded as 5 levels. Our LMM with random slope and random intercept still allows us to quantify the dynamic changes between ITP and CBV (MBV) in the 5 RMs because it has taken the individual differences into account by containing a random slope in ITP. Our model shows that the changes in ITP can predict the changes in MBV (fitted slope is 0.113, p < 0.001), but they cannot predict the changes in CBV (fitted slope is 0.0005, p = 0.965). These results suggest that the changes in MBV during the strains can be partially explained by changes in ITP, but CBV seems to be independent from ITP strains.

### LVSV and HR changes

**Figure 4:**
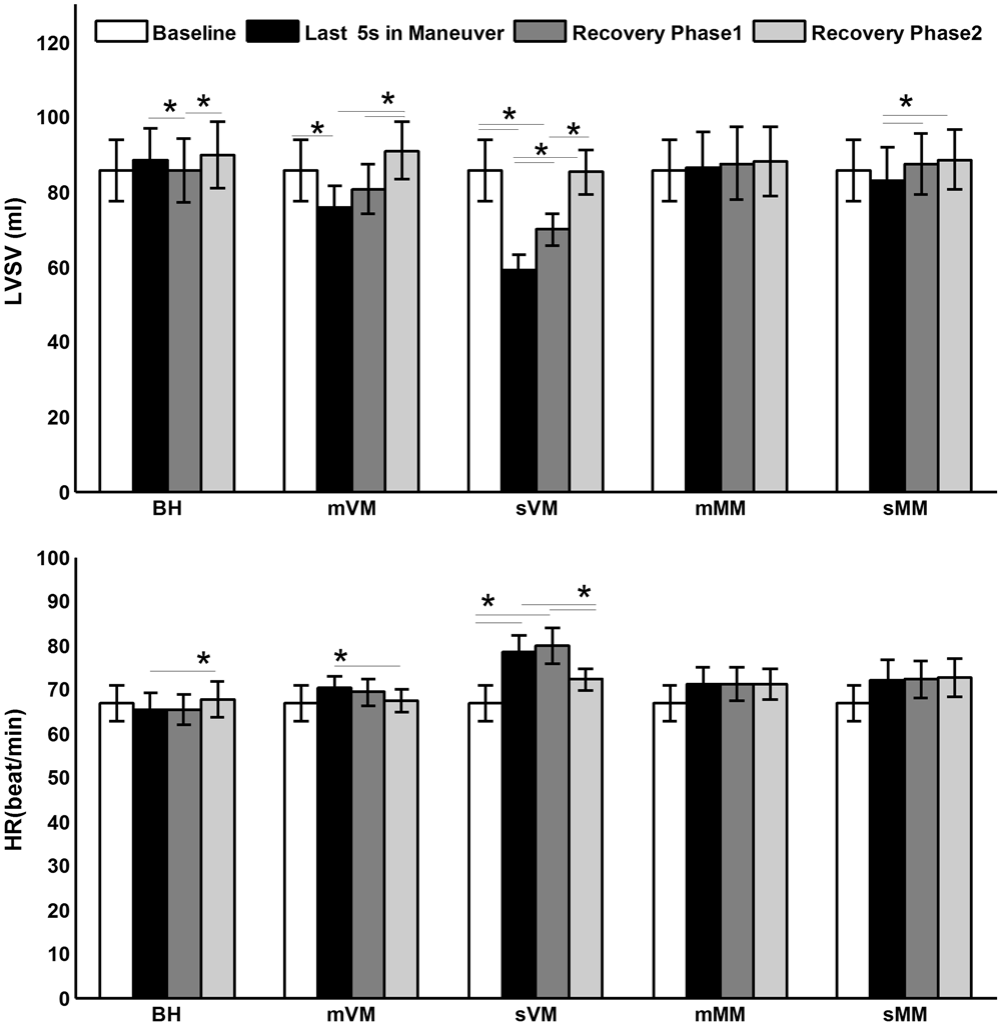
The changes of left ventricular stroke volume (LVSV) and heart rate (HR) in each maneuver protocol. ^*^Indicates significant differences between the two ends of the short lines. Recovery phase 1 is the first 5 s post strain, and recovery phase 2 is the second 5 s of recovery. The error bars are standard error and n = 11.

One-way repeated ANOVA gives significant main effect of changes in LVSV in mVM (F(3,30) = 6.858, p = 0.007) and sVM (F(3,30) = 20.112, p < 0.0001). After BH the mean LVSV falls in the first 5 s of recovery and then increases (Fig. 4). In mVM and sVM, LVSV significantly decreases within the last 5 s of strains and increases post stains. The decrement of LVSV is more profound in sVM compared to mVM (LVSV: 59.15 ± 4.04 ml vs. 75.99 ± 5.6 ml, p = 0.0005). Significant increasing LVSV during recovery phase is found in mVM, sVM and sMM.

Significant main effect of changes in HR is only found in sVM (F(3,30) = 6.28, p = 0.012). HR only significantly increases within the last 5 s of strain in sVM (Fig. 4). During recovery HR increases in BH but decreases in mVM and sVM.

Table 1 below summarizes the changing directions of CBV, MBV, ITP, LVSV and HR in the each RM protocol. There is correlation between MBV and LVSV (r = −0.16, p = 0.04), and between MBV and HR (r = 0.20, p = 0.01). Neither LVSV (r = 0.03, p = 0.71) nor HR (r = −0.08, p = 0.28) correlates to CBV.

**Table 1:** The summary of ITP, CBV, MBV, LVSV and HR changes within the strains and recovery phases.

		ITP	CBV	MBV	LVSV	HR
Stable phase within maneuver	BH	—	—	—	—	—
	mMM	↓	—	↘(p = 0.065, 5 s vs. baseline)	—	—
	sMM	↓	—	↓	—	—
	mVM	↑	—	↑	↓	—
	sVM	↑	—	↑	↓	↑
Recovery	BH	—	↑	↓	↓	↑
	mMM	↑	↑	↖(p = 0.09, 20 s vs. 15 s)	—	—
	sMM	↑	↑	—	↑	—
	mVM	↓	↑	↓	↑	↓
	sVM	↓	↑	↓	↑	↓

## Discussion

We characterize changes in cerebral and peripheral hemodynamics by measuring cerebral and muscular blood volume (CBV and MBV) together with cardiac stroke volume (i.e., LVSV) and heart rate (HR) within the strain and in the recovery phase of 5 graded respiratory maneuvers (RMs) in the same subjects. The changes in intrathoracic pressure (ITP) induced by the RMs are carefully monitored by esophageal manometry. We find that despite of the various changes of ITP within the RMs, CBV is stable compared to its baseline but MBV changes in the same direction as ITP. Cerebral StO_2_ only decreases within strong Valsalva maneuver. In the recovery phase, CBV increases after the release of strain in each RM protocol despite of opposing peripheral parameters associated with the strain, whereas MBV still changes in the same direction as ITP except in breath holding and strong Mueller maneuver. These results suggest that 1). The measured CBV with FDMD NIRS truly reflects BV from the brain and not from peripheral tissues; 2). Intact cerebral autoregulation is able to cope with various ITPs within RMs and capable to maintain an adequate cerebral perfusion and oxygenation, with a special challenge for strong Valsalva maneuver; 3). Increasing CBV is a common phenomenon following the release of different degrees of strain in each RM and is therefore controlled by local mechanisms to increase the cerebral oxygen supply independent of changes in systematic parameters and ITP.

Are our NIRS results reliable? This is the first question we need to answer before further discussion. The superficial contamination is well known in NIRS^26, 27^, raising the question if any further conclusions can be reliably drawn from our results. Currently most of the available NIRS devices are based on CW technique in which hemodynamics is calculated with MBLL. They are contaminated by superficial tissues because light travels much longer distance than the source-detector-distance $d$ due to scattering but it is unable to measure the light travelling path-length in the deep tissue (i.e., it is unable to distinguish the absorption from superficial and deep tissues)^15^. MBLL uses differential path length factor to account for the increased light travelling pathway due to scattering, whose values derive from literatures (i.e., fixed values) but actually vary between subjects^15^. This differential path length factor method also has the problem of inducing cross-talk between the measured HbO_2_ and HHb^15^. Spatially resolved spectroscopy (SRS) enables CW NIRS to measure the absolute value of StO_2_ in brain and muscle via a multi-distance design and differentiating the light attenuation $A$ with respect to $d$ (i.e., $\partial A/\partial d$)^34^.The $\partial A/\partial d$ becomes linear and the photons detected by each channel approximately probe the same volume, given that SRS NIRS has more than two different $d$ and small channel-separation (i.e., the distances between detectors or light sources are small enough to do differential calculus $\partial A/\partial d$). Thus the superficial influence can be subtracted and the hemodynamics calculated with SRS method is more sensitive to deep tissues^15, 34^. In reference^34^ the introduced device (NIRO-300 from Hamamatsu Photonics KK, Japan) has three different $d$ with a separation of 1 mm, and its reliability of measuring cerebral StO_2_ has been validated in patients undergoing carotid endarterectomy^35^. Nevertheless the parameters calculated with MBLL in SRS NIRS measurement are still vulnerable to superficial interference^26, 35^. Cerebral StO_2_ measured by SRS NIRS devices with only two different source-detector distances and larger channel-separation (e.g., more than 1 cm) is also contaminated by extracranial tissues^27^, probably because of the nonlinearity of $\partial A/\partial d$ (i.e., the linearity cannot be assessed with only two source-detector distances) and the light probes different superficial volumes. Therefore, we suggest CW NIRS signals calculated with either MBLL or SRS of two source-detector distances and large channel-separation should be interpreted cautiously; the ones measured with SRS method like NIRO-300 (i.e., more than two source-detector distances, and the channel-separation is small) should be more sensitive to deeper tissues.

We use FDMD NIRS, a different technique that is superior to the CW NIRS because it can measure absorption and reduced scattering of the measured tissue and effectively suppress the superficial contamination (see Method Section 4.3). Its reliability of measuring cerebral hemodynamics has been well validated in previous studies^30–32^. We find that CBV and MBV change differently in our experiment, suggesting that they are measured from different sources. To compare our results with the ones of previous studies, we find that in VM our CBV changes disagree with the ones calculated with MBLL^23, 24, 26^ but agree with the ones calculated with SRS in NIRO-300 recordings^26^. The changes in our cerebral StO_2_ during VM also fit the ones measured with NIRO-200 (an updated version of NIRO-300)^24^. We believe our results of cerebral hemodynamics measured by FDMD NIRS are reliable.

Increasing CBV after the strains of all RMs conflicts our second hypothesis, which may indicate a new character of dynamic CA or be regulated by other mechanisms. This observational study cannot allow us to clarify the underlying physiological mechanisms. But our results plausibly suggest brain specific mechanisms, as we do not find this phenomenon in the muscular hemodynamics (left biceps brachii muscle). The increased CBV can be reasonably explained by a residual vasodilatation as the regulation of CA is not instantaneous, or reflect the intrinsic characteristics of cerebral hyperemic responses^3, 24, 36^. Several other cerebral mechanisms (e.g., neurovascular coupling^37^, cerebrovascular CO_2_ reactivity^38^, astrocytic vasodilating pathway^39^) should also be considered as potential candidates calling for future studies. Our results further suggest that the changes in CBV are not dominated by systemic factors including ITP swings, LVSV and HR because CBV changes homogeneously but the systemic parameters change heterogeneously in partly opposing directions among different RMs. In our results, neither LVSV nor HR correlates to CBV change, and the relative stable CBV within the strains cannot be predicted by the ITP swings. People may argue that the increased CBV could also indicate inability of CA to suppress the increased MAP to maintain a relatively constant cerebral perfusion after strains (i.e., the increased CBV is induced by increased MAP). The change in MAP may influence the CBV but we do not think this is the major factor inducing the increased CBV, although we do not measure MAP. First, recent studies showed that the ability of CA to damp a rise in MAP is greater (approximately twice) than the one to withstand a reduction in MAP^40^. Second, previous studies reported that the MAP after VM and MM increases very quickly (several seconds) and it returns to baseline levels in about 10 seconds^3, 41, 42^. By contrasts we find that the CBV increases slowly for more than 10 seconds and it cannot return to the baseline levels even 20 seconds after the release of strain (Fig. 1). Third, Zhang *et al*. found that the increment in CBF post the VM strain still exists even after the overshoot of blood pressure is abolished by ganglionic blocker trimethaphan in healthy subjects^3^, suggesting that the increasing MAP does not dominate the increasing cerebral perfusion post VM strains.

The uniformly increased CBV post strains can bring more oxygen supply to the brain as we find cerebral HbO_2_ and OI increase. A hypothesis of the functional implication of the increased CBV and vasodilatation is that it may facilitate the washout of the accumulated brain metabolites during the stains. A recent study^43^ showed that inspiration is the major driving force for cerebrospinal fluid (CSF) flux which can clean metabolites from the brain. Cerebral perfusion may influence the metabolite clearance as recent animal study found that chronic cerebral hypoperfusion can compromise the clearance of perivascular amyloid beta thus impairing microvascular function^44^. Therefore, whether the increased CBV is associated with increased CSF flux driven by the restored inspiration is an interesting topic for people in the field of neurology.

Strong increment in ITP is more challenging for the brain. First, cerebral StO_2_ significantly decreases within sVM but remains stable within sMM. Given the relative constant CBV within sVM, the decline of cerebral StO_2_ is due to increased oxygen extraction. The decreased cerebral StO_2_ can partly explain why sometime people have syncope during strong VM. Second, MBV increases within sVM, which can be explained by the pooling of blood in the venous system in muscle. However, this venous pooling effect unlikely exists in the brain as no change is found in CBV. The venous pooling may compromise the CA as more blood is retained in muscular veins. Third, LVSV is much smaller in later phase of sVM compared to sMM (59.15 ± 4.04 ml vs. 83.24 ± 8.7 ml). Although HR significantly increases within sVM, it still may be not sufficient enough to compensate the reduced systemic blood supply.

ITP is a major factor controlling changes in MBV as they change in the same direction except in the recovery phase of BH and sMM. Our results of LMM analysis also suggest that change in ITP in the strain is a strong predictor of the change in MBV. We only find relative week correlations between LVSV and MBV (r = −0.16, p = 0.04), and between HR and MBV (r = 0.20, p = 0.01). ITP may control MBV via mechanically changing the stresses of muscular vessel wall or inducing sympathetic activities in vessels, and thus leading to vasodilatation/vasoconstriction.

Increased MBV within VM suggests that blood is detained in the muscle, thus venous return should decrease. The reduced venous return can decrease preload and thus LVSV decreases. Our data show that the higher the ITP is (mVM vs. sVM), the increment of MBV and decrement of LVSV are more profound. These results suggest that the degree of impaired venous return may be related to the degree of increment in strain. Increased LVSV after VM could be explained by increasing venous return and vasoconstriction in peripheral tissues, because the increment in MBV within RMs could generate high vasoconstriction tone.

Our study suggests that it should be careful to use MM to imitate obstructive sleep apnea event as the CH changes differently. Decreasing CBV after obstructive sleep apnea events was reported in previous studies with NIRS^20, 45^, conflicting our results of increasing CBV after MM. Considering the similar changes in ITP, we hypothesize that hypoxia and/or hypercapnia occurring during obstructive sleep apnea events may account for the differences. Cerebral StO_2_ does not change in the stable phase of MM compared to baseline, indicating no hypoxia within MM; while decreased cerebral StO_2_ has been repeatedly found during obstructive sleep apnea events^20, 21^. We do not measure CO_2_ but it is likely to cumulate during MM because of the block of the exhaling. Another mechanism contributing to the differences is arousal system. In obstructive sleep apnea the termination of apnea event is usually associated with cortical arousal, which can independently increase sympathetic nerve activity and accentuates vasoconstriction^46^.

There are several limitations in our study. First, we do not measure CO_2_ which limits us to discuss the role of CO_2_ reactivity in regulating CH. Recent study in healthy people using pseudo-continuous arterial spin labeling MRI showed that under constant CO_2_ ITP strain alone can induce local perfusion in somatosensory/motor cortices but not in the prefrontal cortex^47^. Our results may suggest that perfusion in forehead may still be stable during ITP strains even with mild hypercapnia. Based on the multi-methodologies approach introduced in this study, future studies adding CO_2_ measurement may further better our knowledge of the dynamic changes in cerebral autoregulation and cerebral circulation under various ITPs. Second, our echocardiography is only performed in the later phase of strain. One of the major limitations of echocardiography in this research field is to obtain clear cardiac signals without movement artifacts induced by the initiations of Valsalva and Mueller maneuvers and the hyperventilation after the release of the ITP strains. Future studies measuring the LVSV and HR within the whole strain may provide more information to interpret changes of cerebral and muscular NIRS signals. Third, we do not measure the MAP. The lack of a quantitative correlation between the observed hemodynamics and objective measurements of MAP limits the ability to specify the physiological origin of the observed hemodynamics. It is suggested that finger photo-plethysmography (Finapres) based on the volume clamp technology is the only unsupervised method for continuous non-invasive blood pressure measurement^48^. But this technology suffers from accuracy and reproducibility problems^49–53^. The Finapres systolic blood pressure measurements do not fulfill the British Hypertension Society nor the Association for the Advancement of Medical Instrumentation standard criteria. Furthermore, very little is known about whether the blood pressure measured with Finapres and intravascular method are equivalent in assessing dynamic CA. A recent study reports significant difference between these two methods in assessing dynamic CA in patients with acute brain injury and the authors suggest invasive arterial blood pressure monitoring should be preferred for dynamic CA assessment^54^. Since in our protocol the ITP swings challenges the dynamic CA, we are uncertain about the accuracy of the Finapres recordings in this scenario and we recommend cautious interpretations of future results assessed by Finapres. In addition, Finapres measures are not feasible in our experimental design as subjects needed to close their noses during the strains with their right hands, and the NIRS muscular sensor was fastened on their left bicep brachii muscle with bandage which hindered the calibration of Finapres using the upper arm cuff.

Nevertheless, our study indicates that FDMD NIRS may provide an economic and easy way to assess the CH under ITP swings. Our results probe the basic physiological connections between cerebral, muscular and cardiac hemodynamic changes under various ITP changes, which may provide insights into cerebral circulation and neurovascular coupling. Our results may also have clinical significances for patients with sleep apnea and for respiratory management in critical care.

## Methods

This study was approved by the local ethical commission of Northwest Switzerland, and was in compliance with the declaration of Helsinki; all subjects gave their written informed consent to participate in the study. The methods were carried out in accordance with the relevant guidelines and regulations.

### Subjects

11 healthy adults (m/f: 6/5; age: 37.3 ± 4.9 yrs; BMI: 23.1 kg/m^2^ (20.8–25.6)) participated in this study. One male subject did the experiment twice in two weeks. The echocardiography failed in his first recording. None of the subjects had any sleep disorders, ischemic heart disease, chronic heart failure, cerebrovascular disorders, hypertension, diabetes, obesity, or any mental health problems.

### Protocol

Every subject did 5 RMs (each RM lasted for 15 s and followed by 20 s recovery phase) in supine position: end expiratory BH, post-inspiration mVM and sVM with increased 20 and 40 cmH_2_O ITP from normal breathing (i.e., expiratory effort against closed upper airway), post-expiratory mMM and sMM with decreased 20 and 40 cmH_2_O ITP from normal breathing (i.e., inspiratory effort against closed upper airway). The subjects closed their mouths and needed to close their noses with the help of their right hands during the maneuvers. The interval between RMs was at least 3 minutes. An adult esophageal balloon catheter (CooperSurgical, Trumbull, CT, USA) was placed by nasal insertion into the esophagus to monitor changes in ITP. The output of the catheter was connected to XLTEK (Natus Neurology, Excel-Tech Ltd., WI, USA) via a dual airflow differential pressure transducer model PT2 Dual (BRAEBON Medical Corporation, Kanata, Ontario, Canada). We provided visual feedback during the entire strain enabling the subjects to adjust and maintain the ITP. All subjects practiced performing the RMs before official recordings.

### Frequency-domain multi-distance (FDMD) near-infrared spectroscopy (NIRS)

FDMD NIRS (Imagent, ISS, Champaign IL, USA) measurements were conducted over the middle of left forehead (i.e., in the middle of the area that below the hairline and above the left brow) and the left bicep brachii muscle. The principle of FDMD NIRS has been well introduced^29, 55^. The major absorbers of near-infrared light in the human tissues were HbO_2_ and HHb. In Imagent system, the light emitters (8 laser diodes, 4 at 690 nm wavelength and 4 at 830 nm wavelength, so they were coupled into 4 sources) were modulated at 110 MHz and the light can penetrate into the measured tissues with a depth of approximate 3–4 cm. The back-scattering light from tissues can be picked up by a 3-mm-diameter optical fiber bundle that was connected to photomultiplier tube detector. To yield a multi-distance measurement, the four coupled light sources were aligned and placed at 2 cm, 2.5 cm, 3 cm and 3.5 cm from the detecting optical fiber bundle. The light intensity (*I*_*DC*_), modulation amplitude (*I*_*AC*_) and phase measured ${\rm{f}}$rom different distances varied linearly (the linearity was monitored by the R^2^ of the fitted linear regression model). Therefore, to submit the measured ${I}_{{DC}}$, ${I}_{{AC}}$ and $phase$ to linear regression we can obtain the following equations^29, 32, 55^

1 $$ln({r}^{2}{I}_{{AC}})=r{S}_{{AC}}\,+{C}_{{AC}}$$

2 $$ln({r}^{2}{I}_{{DC}})=r{S}_{{DC}}\,+{C}_{{DC}}$$

3 $$phase=r{S}_{{\rm{phase}}}\,+{C}_{{\rm{phase}}}$$

where $r$ was the known source-detector distance, *S*_*AC*_, *S*_*DC*_ and *S*_*phase*_ were the slopes and *C*_*AC*_, *C*_*DC*_, *C*_*phase*_ were the intercepts. The superficial influences in each channel can be attributed to the intercepts and the residuals of the linear regression. The cerebral hemodynamic parameters were calculated with the slopes, i.e., to combine any two of these three slopes we can estimate absorption and reduced scattering coefficients of the measured tissue and then to further calculate HbO_2_ and HHb^29^. Then total hemoglobin reflecting BV changes was calculated as the sum of HbO_2_ and HHb, and the ratio of HbO_2_ over total hemoglobin provided an index for changes of StO_2_. Normally people chose$\,{S}_{{AC}}$ and ${S}_{{phase}}$, considering that ${I}_{{AC}}$ and $phase$ were less contaminated by the environment light. The sample rate of our FDMD NIRS recording was set as 10.4 Hz. Before the start of every measurement, the NIRS device was calibrated on optical phantom blocks.

### Echocardiography

Transthoracic echocardiography (Vivid7, GE Healthcare, USA) was performed during the last stable 5 s of RM and in the first 10 s of recovery period to measure LVSV and HR. A 10-s baseline measurement was performed at rest state. LVSV was assessed using pulsed-wave Doppler sampled in the left ventricular outflow tract (LVOT). It was calculated automatically with the default formula (*LVOT*_*Diam*_)^2^ × 0.785 × (*LVOT*_*VTI*_), where *LVOT*_*Diam*_ was the LVOT diameter and *LVOT*_*VTI*_ was the LVOT velocity time integral.

### Data preprocessing

The mean LVSV and HR during 10 s baseline were calculated, as a baseline value for the multiple comparisons of LVSV and HR changes within maneuvers and in the recovery phase. The LVSV and HR during the last 5 s of maneuver were averaged respectively, and the first 10 s of recovery was segmented into two phases (i.e., first 5 s recovery and second 5 s recovery) within which the mean LVSV and HR were calculated. The similar data average and segmentation have been used for characterizing LVSV dynamics in a study of 15-second MM by Bradley *et al*.^6^. It frequently happened that one heart beat pulse was shared by two consecutive segmentations, because we chose a fixed 5 s time window to segment the 15 s continuous LVSV and HR recordings. In this case, those pulses were excluded from the average process. The mean value of the ITP in the last 5 s of the strain period was calculated in each RM. Since the subjects needed to adjust and maintain the ITP level with the help of visual feedback, the ITP values of the first few seconds of the stain were usually unstable thus we treated its values of the last 5 s within the stain as a more stable and reliable recordings to indicate the level of ITP strains.

NIRS signals were averaged every 1 s (i.e., down sample to 1 Hz) to improve the signal to noise ratio. The reliability of FDMD NIRS measurement depended on the linearity of the raw optical signals on distances, i.e., the linear dependence R^2^ of equations (1) and (3) should be highly close to 1^18, 29, 56^. We checked the R^2^ of the regression fit and the p-values for the linearly fitted absorption and reduced scattering coefficients. The raw optical data were discarded if the R^2^ smaller than 0.97 in either modulation amplitude or phase shift^18, 56^. Then the data were further averaged every 5 s to down sample to 0.2 Hz, in order to be better compared with the cardiac and ITP recordings (i.e., echocardiography data were down sampled and averaged every 5 s and the mean ITP of the last 5 s of strain was treated as the stable ITP value achieved by the subject). In each RM, the mean value of 5 s BV recordings before the start of strain was calculated as baseline and was subtracted from following recordings. Then the relative BV changes were normalized to their baselines in each RM in order to make a valid comparison on group level. The same normalization was also done to cerebral StO_2_, HbO_2_, HHb and OI.

### Statistical analysis

Data were expressed as the mean ± standard error (SE) after preprocessing. Linear mixed model (LMM, more specifically, LMM with random slope) was used to fit the changes of CBV and MBV with time (continuous variable) as explanatory variable, respectively. The LMM was applied to each type of RM separately. LMM was also used to investigate if changes of CBV and MBV showed significant difference in the same RM, via adding a categorical variable ‘tissue’ (i.e., brain or muscle) and merging CBV and MBV as the dependent variable into the model.

LMM (LMM with random slope and random intercept) was also used to check if the changes in ITP manipulated by the 5 RMs can predict the corresponding changes in CBV and MBV, respectively. The explanatory variable was the mean ITP values of the last stable 5 s within the strains of the 5 RMs (i.e., the changes of ITP in the 5 RMs were taken together as the explanatory variable, which represented the graded changes of ITP), and the dependent variables were the changes of CBV and MBV in the last 5 s of the strains, respectively. We did not consider the ITP changes during the recovery period, because the strong hyperventilation following the release of the strain can induce movement artifacts in ITP recordings. We compared the mean values of BV changes of every 5 s to their baseline with Paired t-tests. The same tests were also used to assess if BV significantly changed in recovery compared to the last 5 s within RMs, and to compare if the changes in paired CBV and MBV at the same time point were different. One-way repeated ANOVA was performed to test the main effect of changes in LVSV. Degrees of freedom were corrected using Greenhouse-Geisser (or Huynh-Feldt) correction if the Greenhouse-Geisser estimate of sphericity was smaller (or larger) than 0.75, but original degrees of freedom were reported for sake of readability. Fisher’s least significant difference (LSD) post hoc test was performed to do pairwise comparisons on LVSV data. The same One-way repeated ANOVA and post hoc tests were also performed to HR changes.

We presented the results of multiple comparisons without any correction. Whereas the false discovery rate (FDR) of multiple comparisons was controlled by Benjamini–Hochberg procedure^57^, and our interpretation of the results involving in the ones after FDR control (i.e., p-values were adjusted using FDR method). Pearson product-moment correlation coefficient was computed to assess the relationship between the CBV (MBV) and LVSV, between CBV (MBV) and HR, respectively. The statistically significant level was p < 0.05. All signal preprocessing was carried out in MATLAB (The Math-Works, Inc., Natick, MA, USA). All statistical analyses were performed using R.

### Data availability

The datasets generated during and/or analysed during the current study are available from the corresponding author on reasonable request.
